# Diaqua­bis­(pyrazine-2-carboxamide-κ^2^ *N* ^1^,*O*)cobalt(II) dinitrate

**DOI:** 10.1107/S1600536812012573

**Published:** 2012-03-31

**Authors:** Ajay Pal Singh Pannu, Seona Lee, Yongjae Lee

**Affiliations:** a Department of Earth System Sciences, Yonsei University, Seoul 120-749, Republic of Korea

## Abstract

The asymmetric unit of the title complex, [Co(C_5_H_5_N_3_O)_2_(H_2_O)_2_](NO_3_)_2_, contains one half of a Co^II^ cationic unit and a nitrate anion. The entire [Co(C_5_H_5_N_3_O)_2_(H_2_O)_2_]^2+^ cationic unit is completed by the application of inversion symmetry at the Co^II^ site, generating a six-coordinate distorted octa­hedral environment for the metal ion. The chelating pyrazine-2-carboxamide mol­ecules are bound to cobalt *via* N and O atoms, forming a square plane, while the remaining two *trans* positions in the octa­hedron are occupied by two coordinated water mol­ecules.

## Related literature

For the monodentate coordination mode of the pyrazine-2-carboxamide ligand, see: Azhdari Tehrani *et al.* (2010 ▶); Mir Mohammad Sadegh *et al.* (2010 ▶); Goher & Mautner (1999 ▶, 2001 ▶). For the chelating bidentate coordination mode, see: Tanase *et al.* (2008 ▶); Prins *et al.* (2007 ▶); Sekisaki (1973 ▶). For coordination by pyrazine carboxamide moieties, see: Hausmann & Brooker (2004 ▶); Cati & Stoeckli-Evans (2004 ▶).
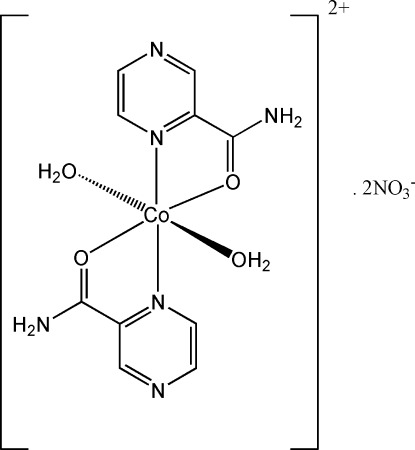


## Experimental

### 

#### Crystal data

[Co(C_5_H_5_N_3_O)_2_(H_2_O)_2_](NO_3_)_2_
*M* *_r_* = 465.22
Monoclinic, 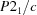

*a* = 10.149 (5) Å
*b* = 6.715 (3) Å
*c* = 13.080 (5) Å
β = 104.397 (4)°
*V* = 863.4 (7) Å^3^
*Z* = 2
Mo *K*α radiation
μ = 1.07 mm^−1^
*T* = 295 K
0.20 × 0.18 × 0.18 mm

#### Data collection

Rigaku R-AXIS IV++ diffractometer
Absorption correction: multi-scan (*CrystalClear*; Rigaku, 2000 ▶) *T*_min_ = 0.815, *T*_max_ = 0.831
4254 measured reflections
1958 independent reflections
1831 reflections with *I* > 2σ(*I*)
*R*_int_ = 0.023

#### Refinement

*R*[*F* ^2^ > 2σ(*F* ^2^)] = 0.033
*wR*(*F* ^2^) = 0.097
*S* = 1.07
1958 reflections
140 parameters
2 restraints
H atoms treated by a mixture of independent and constrained refinement
Δρ_max_ = 0.42 e Å^−3^
Δρ_min_ = −0.55 e Å^−3^

### 

Data collection: *CrystalClear* (Rigaku, 2000 ▶); cell refinement: *CrystalClear*; data reduction: *CrystalClear*; program(s) used to solve structure: *SIR92* (Altomare *et al.*, 1993 ▶); program(s) used to refine structure: *SHELXL97* (Sheldrick, 2008 ▶); molecular graphics: *ORTEP-3* (Farrugia, 1997 ▶); software used to prepare material for publication: *WinGX* (Farrugia, 1999 ▶).

## Supplementary Material

Crystal structure: contains datablock(s) I, global. DOI: 10.1107/S1600536812012573/mw2053sup1.cif

Structure factors: contains datablock(s) I. DOI: 10.1107/S1600536812012573/mw2053Isup2.hkl

Additional supplementary materials:  crystallographic information; 3D view; checkCIF report

## Figures and Tables

**Table 1:** Hydrogen-bond geometry (Å, °)

*D*—H⋯*A*	*D*—H	H⋯*A*	*D*⋯*A*	*D*—H⋯*A*
O1*W*—H1*W*⋯O4^i^	0.82 (1)	1.93 (1)	2.742 (2)	170 (3)
O1*W*—H2*W*⋯O4^ii^	0.82 (1)	1.92 (1)	2.722 (2)	164 (3)

Symmetry codes: (i) 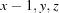; (ii) 
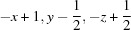
.

**Fig. 1.**
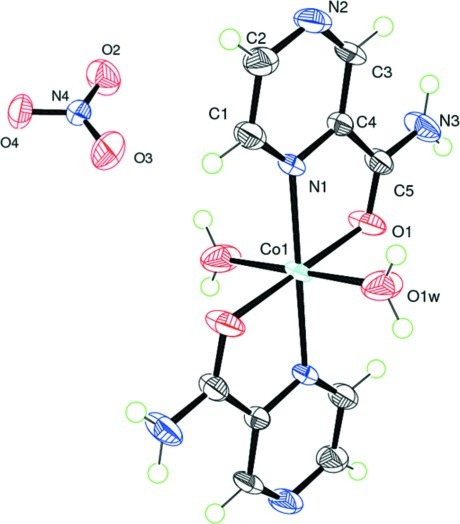
The ORTEP diagram showing the molecular structure of the title complex. The ellipsoids are drawn at the 50% probability level. Unlabelled atoms are related to the labelled atoms by the symmetry transformation (-x, -y, -z + 1) .

**Fig. 2.**
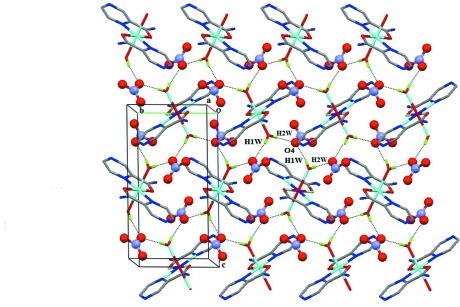
The two dimensional sheet structure parallel to the bc plane is formed by O—H···O hydrogen bonding interactions between the complex cations and the nitrate ions. H-atoms other than those involved in H-bonding have been omitted for clarity. Hydrogen bonds are shown as dashed lines.

## supplementary crystallographic information

### Comment

The ligand pyrazine-2-carboxamide can coordinate to a metal center in a monodentate fashion through the pyrazine nitrogen atom which is *meta* to the carboxamide group. Alternatively, when the ligand uses both the carboxamide oxygen atom and the pyrazine nitrogen atom *ortho* to it for coordination, a stable five member ring is formed as a result of the ligand coordinating in chelating bidentate fashion.

In the present study we report the synthesis, molecular and crystal structure of an octahedral complex of Co^II^ with the pyrazine-2-carboxamide ligand, [Co(C_5_H_5_N_3_O)_2_(H_2_O)_2_](NO_3_)_2_. The molecular structure of this complex is shown in Fig. 1. In this complex, the Co^II^ atom lies on a center of inversion and adopts an octahedral geometry. Two pyrazine-2-carboxamide ligand molecules, each coordinating to the Co^II^ center in a chelating bidentate fashion and forming a stable five membered ring, form a square planar arrangement around the metal center. The remaining two *trans* positions in the octahedron are occupied by two coordinated water molecules. The crystal packing is dominated by O—H···O hydrogen bonding interactions between the complex molecules and the nitrate ions present in the crystal lattice which leads to the formation of a two-dimensional sheet parallel to the *bc* plane (Fig. 2, Table 1).

### Experimental

A solution of pyrazine-2-carboxamide (0.246 g, 2.0 mmol) in ethanol (10 ml) was added to a solution of cobalt(II) nitrate hexahydrate (0.291 g, 1.0 mmol) in water (5 ml) at room temperature. After stirring the resulting solution for 3–4 h, an orange colored solid had formed which was filtered off and dried. Orange crystals of the title complex were obtained by slow evaporation from acetonitrile solution over two weeks.

### Refinement

All non hydrogen atoms were refined anisotropically. The hydrogen atoms of the coordinated water molecules were located from the Fourier difference maps and included as riding contributions with O—H distances set to 0.82 Å with *U*_iso_(H) = 1.2*U*_eq_(*O*). All other H atoms were positioned geometrically with C–H = 0.93 and N—H = 0.86 Å and constrained to ride on their parent atoms, with *U*_iso_(H) = 1.2*U*_eq_(C,*N*).

### Crystal data

**Table:**

[Co(C_5_H_5_N_3_O)_2_(H_2_O)_2_](NO_3_)_2_	*F*(000) = 474
*M**_r_* = 465.22	*D*_x_ = 1.789 Mg m^−^^3^
Monoclinic, *P*2_1_/*c*	Mo *K*α radiation, λ = 0.71069 Å
Hall symbol: -P 2ybc	Cell parameters from 62 reflections
*a* = 10.149 (5) Å	θ = 1.6–30.1°
*b* = 6.715 (3) Å	µ = 1.07 mm^−^^1^
*c* = 13.080 (5) Å	*T* = 295 K
β = 104.397 (4)°	Block, orange
*V* = 863.4 (7) Å^3^	0.2 × 0.18 × 0.18 mm
*Z* = 2	

### Data collection

**Table:**

Rigaku R-AXIS IV++ diffractometer	1958 independent reflections
Confocal monochromator	1831 reflections with *I* > 2σ(*I*)
Detector resolution: 10 pixels mm^-1^	*R*_int_ = 0.023
φ scans	θ_max_ = 30.1°, θ_min_ = 1.6°
Absorption correction: multi-scan (*CrystalClear*; Rigaku, 2000)	*h* = −13→14
*T*_min_ = 0.815, *T*_max_ = 0.831	*k* = −7→9
4254 measured reflections	*l* = −13→18

### Refinement

**Table:**

Refinement on *F*^2^	Secondary atom site location: difference Fourier map
Least-squares matrix: full	Hydrogen site location: inferred from neighbouring sites
*R*[*F*^2^ > 2σ(*F*^2^)] = 0.033	H atoms treated by a mixture of independent and constrained refinement
*wR*(*F*^2^) = 0.097	*w* = 1/[σ^2^(*F*_o_^2^) + (0.0597*P*)^2^ + 0.1848*P*] where *P* = (*F*_o_^2^ + 2*F*_c_^2^)/3
*S* = 1.07	(Δ/σ)_max_ < 0.001
1958 reflections	Δρ_max_ = 0.42 e Å^−^^3^
140 parameters	Δρ_min_ = −0.55 e Å^−^^3^
2 restraints	Extinction correction: *SHELXL*
Primary atom site location: structure-invariant direct methods	Extinction coefficient: 0.058 (5)

### Special details

**Table:**

Geometry. All e.s.d.'s (except the e.s.d. in the dihedral angle between two l.s. planes) are estimated using the full covariance matrix. The cell e.s.d.'s are taken into account individually in the estimation of e.s.d.'s in distances, angles and torsion angles; correlations between e.s.d.'s in cell parameters are only used when they are defined by crystal symmetry. An approximate (isotropic) treatment of cell e.s.d.'s is used for estimating e.s.d.'s involving l.s. planes.
Refinement. Refinement of *F*^2^ against ALL reflections. The weighted *R*-factor *wR* and goodness of fit *S* are based on *F*^2^, conventional *R*-factors *R* are based on *F*, with *F* set to zero for negative *F*^2^. The threshold expression of *F*^2^ > σ(*F*^2^) is used only for calculating *R*-factors(gt) *etc*. and is not relevant to the choice of reflections for refinement. *R*-factors based on *F*^2^ are statistically about twice as large as those based on *F*, and *R*- factors based on ALL data will be even larger.

### Fractional atomic coordinates and isotropic or equivalent isotropic displacement parameters (Å^2^)

**Table:**

	*x*	*y*	*z*	*U*_iso_*/*U*_eq_	
C1	−0.13028 (17)	0.3679 (3)	0.37319 (14)	0.0252 (4)	
H1	−0.045	0.4279	0.3917	0.03*	
C2	−0.2368 (2)	0.4620 (3)	0.30214 (17)	0.0312 (4)	
H2	−0.2209	0.5835	0.2733	0.037*	
C3	−0.38018 (17)	0.2122 (3)	0.32023 (14)	0.0265 (4)	
H3	−0.4668	0.1565	0.305	0.032*	
C4	−0.27504 (15)	0.1155 (2)	0.38998 (12)	0.0187 (3)	
C5	−0.28494 (16)	−0.0812 (3)	0.44282 (13)	0.0233 (4)	
N1	−0.14921 (13)	0.1933 (2)	0.41483 (10)	0.0190 (3)	
N2	−0.36101 (16)	0.3835 (3)	0.27411 (13)	0.0328 (4)	
N3	−0.40489 (16)	−0.1673 (3)	0.42833 (14)	0.0338 (4)	
H3A	−0.4122	−0.2795	0.4581	0.041*	
H3B	−0.4758	−0.1112	0.3891	0.041*	
N4	0.73095 (19)	−0.0030 (2)	0.13278 (13)	0.0257 (4)	
O1	−0.17918 (12)	−0.1555 (2)	0.49866 (11)	0.0307 (3)	
O2	0.67682 (16)	−0.1413 (2)	0.16811 (14)	0.0475 (4)	
O3	0.67169 (18)	0.0854 (3)	0.05233 (14)	0.0574 (5)	
O4	0.85161 (15)	0.0470 (2)	0.18035 (12)	0.0361 (3)	
Co1	0	0	0.5	0.01880 (16)	
O1W	0.00264 (16)	−0.1345 (2)	0.35909 (11)	0.0400 (4)	
H2W	0.059 (2)	−0.215 (3)	0.349 (2)	0.048*	
H1W	−0.045 (2)	−0.094 (4)	0.3028 (11)	0.048*	

### Atomic displacement parameters (Å^2^)

**Table:**

	*U*^11^	*U*^22^	*U*^33^	*U*^12^	*U*^13^	*U*^23^
C1	0.0174 (8)	0.0248 (9)	0.0307 (9)	−0.0021 (6)	0.0005 (7)	0.0021 (6)
C2	0.0257 (10)	0.0232 (8)	0.0405 (11)	0.0023 (7)	−0.0002 (8)	0.0086 (8)
C3	0.0129 (8)	0.0289 (9)	0.0322 (9)	0.0019 (6)	−0.0047 (7)	0.0007 (7)
C4	0.0136 (7)	0.0203 (8)	0.0204 (7)	0.0010 (6)	0.0008 (6)	−0.0017 (6)
C5	0.0165 (8)	0.0267 (9)	0.0240 (8)	−0.0016 (7)	0.0000 (6)	0.0020 (6)
N1	0.0124 (6)	0.0228 (7)	0.0194 (6)	0.0017 (5)	−0.0008 (5)	0.0004 (5)
N2	0.0210 (8)	0.0292 (8)	0.0409 (9)	0.0047 (6)	−0.0061 (7)	0.0078 (6)
N3	0.0166 (7)	0.0363 (9)	0.0426 (9)	−0.0074 (6)	−0.0040 (6)	0.0115 (7)
N4	0.0229 (9)	0.0313 (9)	0.0223 (8)	0.0009 (5)	0.0041 (7)	0.0009 (5)
O1	0.0150 (6)	0.0337 (7)	0.0383 (7)	−0.0011 (5)	−0.0027 (5)	0.0143 (6)
O2	0.0453 (9)	0.0434 (9)	0.0557 (10)	−0.0129 (7)	0.0163 (8)	0.0063 (7)
O3	0.0407 (9)	0.0808 (14)	0.0426 (9)	0.0083 (9)	−0.0048 (7)	0.0283 (9)
O4	0.0270 (8)	0.0405 (8)	0.0351 (8)	−0.0068 (6)	−0.0031 (6)	0.0039 (6)
Co1	0.0103 (2)	0.0237 (2)	0.0192 (2)	0.00234 (10)	−0.00225 (14)	0.00248 (10)
O1W	0.0415 (9)	0.0490 (9)	0.0237 (7)	0.0229 (7)	−0.0031 (6)	−0.0045 (6)

### Geometric parameters (Å, º)

**Table:**

C1—N1	1.327 (2)	N3—H3A	0.86
C1—C2	1.390 (3)	N3—H3B	0.86
C1—H1	0.93	N4—O2	1.226 (2)
C2—N2	1.330 (3)	N4—O3	1.227 (2)
C2—H2	0.93	N4—O4	1.273 (2)
C3—N2	1.335 (3)	O1—Co1	2.0934 (14)
C3—C4	1.382 (2)	Co1—O1W	2.0586 (15)
C3—H3	0.93	Co1—O1W^i^	2.0586 (15)
C4—N1	1.343 (2)	Co1—O1^i^	2.0934 (14)
C4—C5	1.505 (2)	Co1—N1^i^	2.0931 (14)
C5—O1	1.243 (2)	O1W—H2W	0.820 (2)
C5—N3	1.318 (2)	O1W—H1W	0.820 (2)
N1—Co1	2.0931 (14)		
			
N1—C1—C2	120.52 (16)	O2—N4—O3	121.3 (2)
N1—C1—H1	119.7	O2—N4—O4	118.83 (18)
C2—C1—H1	119.7	O3—N4—O4	119.88 (17)
N2—C2—C1	122.04 (18)	C5—O1—Co1	115.20 (11)
N2—C2—H2	119	O1W—Co1—O1W^i^	180
C1—C2—H2	119	O1W—Co1—O1^i^	91.24 (7)
N2—C3—C4	121.87 (16)	O1W^i^—Co1—O1^i^	88.76 (7)
N2—C3—H3	119.1	O1W—Co1—O1	88.76 (7)
C4—C3—H3	119.1	O1W^i^—Co1—O1	91.24 (7)
N1—C4—C3	120.57 (15)	O1^i^—Co1—O1	180
N1—C4—C5	113.48 (13)	O1W—Co1—N1	87.95 (6)
C3—C4—C5	125.94 (15)	O1W^i^—Co1—N1	92.05 (6)
O1—C5—N3	122.68 (17)	O1^i^—Co1—N1	101.95 (6)
O1—C5—C4	118.41 (14)	O1—Co1—N1	78.05 (6)
N3—C5—C4	118.91 (15)	O1W—Co1—N1^i^	92.05 (6)
C1—N1—C4	118.09 (14)	O1W^i^—Co1—N1^i^	87.95 (6)
C1—N1—Co1	127.39 (11)	O1^i^—Co1—N1^i^	78.05 (6)
C4—N1—Co1	113.95 (11)	O1—Co1—N1^i^	101.95 (6)
C2—N2—C3	116.81 (16)	N1—Co1—N1^i^	180
C5—N3—H3A	120	Co1—O1W—H2W	127 (2)
C5—N3—H3B	120	Co1—O1W—H1W	122 (2)
H3A—N3—H3B	120	H2W—O1W—H1W	110 (3)
			
N1—C1—C2—N2	0.8 (3)	N3—C5—O1—Co1	175.70 (14)
N2—C3—C4—N1	0.9 (3)	C4—C5—O1—Co1	−4.4 (2)
N2—C3—C4—C5	−177.61 (17)	C5—O1—Co1—O1W	−81.16 (14)
N1—C4—C5—O1	−3.1 (2)	C5—O1—Co1—O1W^i^	98.84 (14)
C3—C4—C5—O1	175.52 (17)	C5—O1—Co1—N1	7.01 (13)
N1—C4—C5—N3	176.79 (15)	C5—O1—Co1—N1^i^	−172.99 (13)
C3—C4—C5—N3	−4.6 (3)	C1—N1—Co1—O1W	−90.46 (15)
C2—C1—N1—C4	−2.9 (2)	C4—N1—Co1—O1W	80.57 (12)
C2—C1—N1—Co1	167.77 (14)	C1—N1—Co1—O1W^i^	89.54 (15)
C3—C4—N1—C1	2.1 (2)	C4—N1—Co1—O1W^i^	−99.43 (12)
C5—C4—N1—C1	−179.19 (14)	C1—N1—Co1—O1^i^	0.38 (15)
C3—C4—N1—Co1	−169.81 (13)	C4—N1—Co1—O1^i^	171.41 (11)
C5—C4—N1—Co1	8.89 (17)	C1—N1—Co1—O1	−179.62 (15)
C1—C2—N2—C3	2.2 (3)	C4—N1—Co1—O1	−8.60 (11)
C4—C3—N2—C2	−3.0 (3)		

Symmetry code: (i) −*x*, −*y*, −*z*+1.

### Hydrogen-bond geometry (Å, º)

**Table:**

*D*—H···*A*	*D*—H	H···*A*	*D*···*A*	*D*—H···*A*
O1*W*—H1*W*···O4^ii^	0.82 (1)	1.93 (1)	2.742 (2)	170 (3)
O1*W*—H2*W*···O4^iii^	0.82 (1)	1.92 (1)	2.722 (2)	164 (3)

Symmetry codes: (ii) *x*−1, *y*, *z*; (iii) −*x*+1, *y*−1/2, −*z*+1/2.
